# Mental Disorders and Their Association With Disability Among Internally Displaced Persons and Returnees in Georgia

**DOI:** 10.1002/jts.21949

**Published:** 2014-10-16

**Authors:** Nino Makhashvili, Ivdity Chikovani, Martin McKee, Jonathan Bisson, Vikram Patel, Bayard Roberts

**Affiliations:** 1 Global Initiative on Psychiatry-TbilisiTbilisi, Georgia; 2 Ilia State UniversityTbilisi, Georgia; 3 Curatio International FoundationsTbilisi, Georgia; 4 European Centre on Health of Societies in Transition, London School of Hygiene and Tropical MedicineLondon, United Kingdom; 5 Cardiff University School of Medicine and Cardiff and Vale University Health BoardCardiff, United Kingdom; 6 Centre for Global Mental Health, London School of Hygiene and Tropical MedicineLondon, United Kingdom

## Abstract

There remains limited evidence on comorbidity of mental disorders among conflict-affected civilians, particularly internally displaced persons (IDPs) and former IDPs who have returned to their home areas (returnees). The study aim was to compare patterns of mental disorders and their influence on disability between IDPs and returnees in the Republic of Georgia. A cross-sectional household survey was conducted with adult IDPs from the conflicts in the 1990s, the 2008 conflict, and returnees. Posttraumatic stress disorder (PTSD), depression, anxiety, and disability were measured using cut scores on Trauma Screening Questionnaire, Patient Health Questionnaire 9, Generalised Anxiety Disorder 7, and the WHO Disability Assessment Schedule 2.0. Among the 3,025 respondents, the probable prevalence of PTSD, depression, anxiety, and comorbidity (>1 condition) was 23.3%, 14.0%, 10.4%, 12.4%, respectively. Pearson correlation coefficients (*p* < .001) were .40 (PTSD with depression), .38 (PTSD with anxiety), and .52 (depression with anxiety). Characteristics associated with mental disorders in regression analyses included displacement (particularly longer-term), cumulative trauma exposure, female gender, older age, poor community conditions, and bad household economic situation; coefficients ranged from 1.50 to 3.79. PTSD, depression, anxiety, and comorbidity were associated with increases in disability of 6.4%, 9.7%, 6.3%, and 15.9%, respectively. A high burden of psychiatric symptoms and disability persist among conflict-affected persons in Georgia.

It is well recognised that populations affected by armed conflict are frequently exposed to traumatic events and daily stressors and at risk of elevated levels of mental health disorders (Miller & Rasmussen, 2010; Steel et al., 2009). Studies suggest that levels of posttraumatic stress disorder (PTSD) recede over time (Steel et al., 2009), but the evidence remains sparse—particularly for refugees and IDPs who have returned to their home areas.

There is also limited evidence on comorbidity between mental disorders among conflict-affected populations in low- and middle-income countries, despite evidence from other settings showing high levels of comorbidity between PTSD, depression, and anxiety (Ayazi, Lien, Eide, Ruom, & Hauff, 2012; O'Donnell, Creamer, & Pattison, 2004). Moreover, there is a need for more evidence on the impact of the mental disorders on broader emotional, social, and economic functioning of persons affected by armed conflict (Blanchet & Roberts, 2013). The Republic of Georgia has experienced two main phases of conflict in recent years, each involving secessionist movements. The first was in the early 1990s, in the regions of Abkhazia and South Ossetia, with fighting leading to the forced displacement of approximately 300,000 people, of whom approximately 200,000 remain as IDPs. The second was in August 2008, when conflict broke out between Georgia and the Russian Federation over South Ossetia, leading to at least 128,000 ethnic Georgians being displaced as IDPs, of whom up to 100,000 have now returned to their home areas in the border region with South Ossetia (returnees; Internal Displacement Monitoring Centre, 2012). The majority of current IDPs live in government-established IDP settlements/villages; some remain in makeshift settlements in former hotels, schools, factories, and hospitals (particularly those displaced from the 1990s conflicts). Governmental, nongovernmental, and United Nations agencies have provided social assistance to IDPs. Their communities, however, are characterised by poor living conditions, high unemployment, poverty, limited integration with local communities, and low access to health care (WHO/Children of Georgia, 2009). Many of the former IDPs from the 2008 conflict who have returned to their home villages (returnees) in the border region with South Ossetia also experience poor living conditions and greater vulnerability to future armed violence (United Nations Security Council, 2009).

There is surprisingly little information on the mental health of these groups and its determinants. Although qualitative assessments have been conducted, only a single epidemiological study appears to have been conducted and this was limited to elderly (≥ 60 years) IDPs and did not examine levels of comorbidity or adjusted risk factors (Johns Hopkins Bloomberg School of Public Health/Institute for Policy Studies, 2012).

The aim of this study was to compare patterns of mental disorders and their influence on disability between IDPs and returnees in the Republic of Georgia. The specific objectives were to examine (a) the presumed prevalence of the mental disorders of PTSD, depression, anxiety, and their comorbidity; (b) the influence of displacement status, time, trauma exposure, and demographic and socioeconomic characteristics on mental disorders; and (c) the influence of the mental disorders and their comorbidity on disability and how this varied by displacement status.

## Method

### Participants

The study used a cross-sectional survey design and multistage random sampling, with stratification by region and displacement status. A total sample size of 3,600 men and women aged ≥18 years was determined to meet the statistical requirements of the overall study. This consisted of 1,200 respondents from each of the three main conflict-affected populations in Georgia: those displaced as a result of conflicts in the 1990s (1990s IDPs), those displaced after the 2008 conflict (2008 IDPs), and former 2008 IDPs who have returned to their home areas after being displaced due to the 2008 conflict (returnees).

Primary sampling units (*n* = 360; 120 per population group) were selected based on probability proportion to size method using a sampling frame of formal and informal IDP settlement population sizes throughout Georgia provided by the Ministry of Internally Displaced Persons, and lists of villages in the border region with South Ossetia provided by the governor's office in the Shida Kartli region. Within each primary sampling unit, the random walk method was used to randomly select households. Within the selected household one person was selected to be interviewed (based on nearest birthday). If there was no response at the household after three visits (on different days and at different times), the next household on the route was visited, with the same process used for refusals or interrupted interviews to ensure the desired sample size. For the purposes of this study, the overall sample (*N* = 3,600) was restricted to only 1990s IDPs or 2008 IDPs or 2008 returnees, with respondents who had been displaced from both the 1990s and 2008 conflicts excluded (*n* = 256) as well as those who reported that they had never been displaced (*n* = 319). The final sample size was therefore 3,025 with a response rate of 84.0%. The sample respondent characteristics, by displacement status, are shown in Table1. Overall, around two thirds of respondents were women, reflecting findings of studies of the general population in Georgia as many men have left to find employment elsewhere (Caucasus Research Resource Centers, 2010).

**Table 1:** Sample Characteristics by Group

	1990s Displaced		2008 Displaced		Returnees	
	*n* = 1,193		*n* = 996		*n* = 836	
Variable	*n*	%	*n*	%	*n*	%
Sex						
Men	414	34.7	331	33.2	275	32.9
Women	779	65.3	665	66.8	561	67.1
Age (years)						
18–39	438	36.7	430	43.2	291	34.8
40–59	418	35.0	300	30.1	321	38.4
60+	337	28.3	266	26.7	224	26.8
Marital status						
Married/cohabiting	640	53.6	716	71.8	571	68.3
Single	319	26.7	148	14.9	132	15.8
Widowed	234	19.7	132	13.3	133	15.9
Education status						
Higher education	301	25.2	204	20.4	130	15.6
Secondary school	808	67.8	671	67.4	632	75.5
Primary/some secondary	84	7.0	121	12.2	74	8.9
Employment status						
Fully employed/self-employed	194	18.4	187	21.1	114	15.7
Irregular paid work	42	4.0	35	4.0	4	0.6
Farmer	3	0.3	3	0.3	127	17.5
Unemployed	397	37.5	219	24.7	141	19.4
Housewife	127	12.0	203	22.9	137	18.9
Retired	294	27.8	239	27.0	202	27.9
Household economic status						
Very good	4	0.3	3	0.3	3	0.4
Good	17	1.4	25	2.5	10	1.2
Average	539	45.3	533	53.6	332	39.7
Bad	406	34.0	346	34.7	356	42.5
Very bad	227	19.0	89	8.9	135	16.2
Trauma exposure						
Lack of shelter	532	44.6	471	47.3	302	36.1
Serious injury	251	21.0	132	13.3	98	11.7
In combat situation	585	49.0	476	47.8	290	34.7
Abducted	23	1.9	12	1.2	4	0.5
Tortured	23	1.9	14	1.4	3	0.4
Witnessed murder, violence against known other	396	33.2	172	17.3	56	6.7
Witnessed murder, violence against stranger	127	10.7	49	4.9	13	1.6
Death of known other during conflict/displacement	487	40.8	185	18.6	104	12.4
Number of events						
None	257	21.5	261	26.2	311	37.2
1	262	22.0	282	28.3	270	32.3
2	227	19.0	241	24.2	183	21.9
3+	447	37.5	212	21.3	72	8.6

Note: *N* = 3,025.

### Procedure

Data collection took place between October and December 2011. The questionnaires were administered by trained and experienced professional fieldworkers through face-to-face interviews in the respondents’ homes, with all interviews conducted in Georgian. All respondents provided informed consent prior to their inclusion in the study. Full respondent anonymity was assured. Exclusion criteria included people deemed under the influence of alcohol or drugs, and those with severe intellectual or mental impairment using predefined criteria related to understanding, expression, communication, and behaviour. Ethics approval was provided by the National Council on Bioethics in Georgia and the London School of Hygiene and Tropical Medicine.

### Measures

PTSD was measured using the Trauma Screening Questionnaire (TSQ), which consists of 10 items on PTSD symptoms over the past 1 week, with 0 = *No* and 1 = *Yes* responses, which are summed to produce an overall score range of 0 to 10, with TSQ's cutoff of ≥ 6 used to indicate possible PTSD (Brewin et al., 2002). Depression was measured using the Patient Health Questionnaire (PHQ-9), which consists of nine questions on depression symptoms over the last 2 weeks, with responses of 0 = *not at all*, 1 = *several days*, 2 = *more than half the days*, and 3 = *nearly every day*, with item scores summed to produce a total score range of 0 to 27, with the PHQ-9's suggested cutoff of ≥ 10 used to indicate at least moderate depression (Kroenke, Spitzer, & Williams, 2001). Anxiety was measured using the Generalised Anxiety Disorder 7 (GAD-7) instrument, which consists of seven questions on anxiety symptoms over the last 2 weeks, with the same response options and scoring as the PHQ-9, which produces a total score range of 0 to 21, with the GAD-7's suggested cutoff of ≥ 10 used to indicate at least moderate anxiety (Spitzer, Kroenke, Williams, & Lowe, 2006). Functioning was assessed using the WHO Disability Assessment Schedule (WHODAS 2.0; 12-item version), which consists of 12 items on six activity domains for functional disability (cognition, mobility, self-care, getting along, life activities, participation) with a recall period of the previous 30 days, with response option scores ranging from 0 = *none* to 4 = *severe*, which are recoded to produce a general disability score that is converted from a score range of 0 to 36 to 0 to 100 (with higher scores representing higher levels of disability; Üstün, Chatterji et al., 2010; Üstün, Kostanjsek, Chatterji, & Rehm, 2010). Instrument translation used standard procedures involving (a) translation from English into Georgian using professional translators, with translations reviewed by Georgian mental health experts individually and then as a group for cultural relevance, content and concept consistency, clarity, and understanding; (b) a back-translation to check for accuracy, consistency, and equivalence, with adjustments made accordingly; and (c) piloting and field testing to refine the instruments further (Mollica, Massagli, & Silove, 2004; van Ommeren et al., 1999).

In this study, the TSQ, PHQ-9, GAD-7, and WHODAS 2.0 showed good internal reliability, with Cronbach's α scores of .86, .86, .90, and .91, respectively. We also conducted a separate pilot survey of 110 randomly selected IDPs living in Tbilisi to assess the instruments’ test-retest reliability by administering the TSQ, PHQ-9, GAD-7, and WHODAS 2.0 to the same respondents 4 days apart, and the intraclass correlation results for them were .97, .98, .96, and .98, respectively (with scores above .80 indicating excellent agreement between the two periods; Bartko, 1966). The TSQ, PHQ-9, GAD-7, and WHODAS 2.0 also showed good validity. For example, for known groups validity, higher levels of exposure to traumatic events correlates with higher levels of disorders (see below); interinstrument correlations (see results of Pearson's test for correlation below); and construct validity, with the PHQ-9 and GAD-7 each showing a single eigenvalue of >1 indicating a single construct. The TSQ showed two eigenvalues >1 that related to the two constructs in the TSQ of reexperiencing and arousal (Brewin et al., 2002).

The main survey questionnaire also contained items on exposure to a range of violent and traumatic events adapted from the Harvard Trauma Questionnaire, which was designed to measure experiences of violent and traumatic events among civilian populations in a range of cultural settings (Mollica et al., 1992), with items selected that were deemed most pertinent to the Georgian context (see Table1 for selected items). A history of displacement was recorded (current displacement status and when displaced). A range of demographic and socioeconomic characteristics were also recorded, including sex, age, education level, marital status, general living conditions, and conditions in the community (each with five response options ranging from *very satisfactory* to *very unsatisfactory* that were condensed into *satisfactory/very satisfactory*, *neither satisfactory/not satisfactory*, and *unsatisfactory/very unsatisfactory* to ensure sufficient statistical power for the statistical analysis), employment status, household assets, and household economic situation (with five response options ranging from *very bad* to *very good* that was condensed into *very good/good/average* versus *bad/very bad* to ensure sufficient statistical power for the statistical analysis).

### Data Analysis

Descriptive analysis was conducted on the sample characteristics and the prevalence of the three mental health conditions, of having any of the three conditions (i.e., ≥1 condition), comorbidity of more than one condition, having all three conditions, and having a single condition with no comorbidity. Pearson's correlation coefficients among the three conditions were also calculated.

Multivariate logistic regression analysis was then used to examine the association of displacement status and time, trauma exposure, and demographic and socioeconomic characteristics with outcomes of PTSD, depression and anxiety, any condition (i.e., ≥ 1 condition) and comorbidity (i.e., >1 condition). Exploratory bivariate analysis was initially conducted with the outcome of any condition and a stepwise approach used to select variables in the final model, which remained statistically significant (*p* < .05). The same variables were then used in separate models for PTSD, depression, anxiety, and comorbidity to examine any differences between the disorders. In this regression analysis, the data were weighted to reflect actual proportions of 1990s IDPs, 2008 IDPs, and returnees in the overall conflict-affected population of Georgia, based upon the sampling frames noted above.

To examine the association of the sets of symptoms and their comorbidity on functional disability, separate regression models were run for each, and adjusted for displacement status, sex, age, and having a long-term illness, health problem, or disability, which evidence has shown are strongly related to disability (Üstün, Chatterji et al., 2010). The WHODAS 2.0 functional disability outcome is a continuous measure, with the β coefficient results representing equivalent changes in the WHODAS 2.0 scoring range of 0 to 100 following the instrument guidelines. The analyses adjusted for the cluster sampling design. All statistical analyses were performed using Stata 13.1 (StataCorp, 2013).

## Results

**Figure 1:**
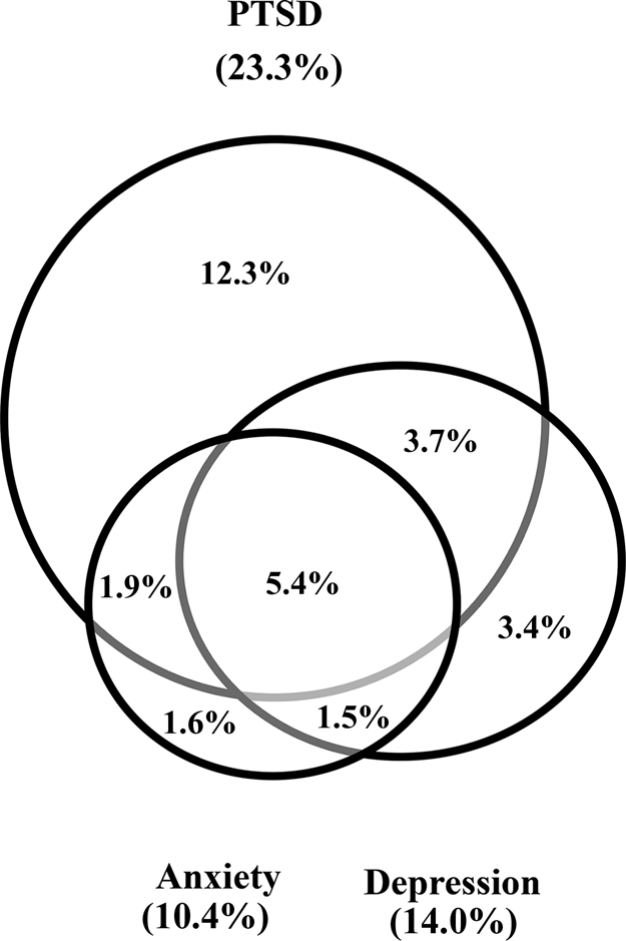
Proportion of respondents with single disorders and with comorbidity (*N* = 3,025).

The proportion of respondents for the combined sample (*N* = 3,025) with the presumed mental disorders and with comorbidity is shown in Figure 1. For this combined sample, the levels were 23.3%, 95% confidence interval (CI) [21.76, 24.80] for PTSD, 14.0%, 95% CI [12.76, 15.24] for depression, and 10.4%, 95% CI [9.39, 11.56] for anxiety. Nearly a third of the combined sample reported at least one condition, 29.44%, 95% CI [27.81, 31.06], and 12.4%, 95% CI [11.24, 13.61] reported more than one, and 5.4%, 95% CI [4.59, 6.21] were above the cut off for all three. When limited to only respondents who had any mental health problem above the cut off, this equated to 41.5%, 95% CI [38.23, 44.79] having a second, and 18.3%, 95% CI [15.76, 20.92] above the cut on all three. There were significant (*p* < .001) correlations between the three scores, with a Pearson correlation coefficient of .40 for PTSD with depression, .38 for PTSD with anxiety, and .52 for depression with anxiety. At between .30 and .60, these can be considered moderate levels of correlation (Hinkle, Jurs, & Wiersma, 1988).

There were significantly higher mean scores (*t* tests, *p* < .001) for the 1990s IDPs compared with the 2008 IDPs and returnees for PTSD (3.53, 3.14, 2.49, respectively), depression (5.43, 3.62, 3.82, respectively), and anxiety (4.42, 3.56, 3.34, respectively). The only significant difference between the mean scores for 2008 IDPs and returnees was for PTSD (*p* < .001).

When using the instrument cutoffs, there were significantly higher levels among 1990s IDPs than returnees for all three sets of symptoms, and also significantly higher levels of depression and comorbidity for 1990s IDPs than for the 2008 IDPs (Table2). Levels of comorbidity for all three conditions were also significantly higher among the 1990s IDPs, 7.3%, 95% CI [5.81, 8.77], than for the 2008 IDPs, 3.8%, 95% CI [2.62, 5.01], and the returnees, 2.6%, 95% CI [1.54, 3.72].

**Table 2:** Prevalence of Presumed Mental Disorders and Comorbidity by Population Group

	1990s Internally			2008 Internally					
	displaced persons			displaced persons			Returnees		
Variable	*n*	%	95% CI	*n*	%	95% CI	*n*	%	95% CI
PTSD	317	27.1	[23.5, 31.1]	226	22.9	[18.9, 27.4]	140	17.0	[13.8, 20.8]
Depression	223	18.7	[15.5, 22.4]	99	9.9	[7.8, 12.6]	60	7.2	[5.3, 9.6]
Anxiety	155	13.0	[10.4, 16.1]	92	9.2	[7.3, 11.7]	55	6.6	[4.3, 10.0]
Any condition	415	34.8	[30.7, 39.1]	282	28.3	[24.2, 32.9]	173	20.7	[17.1, 24.8]
>1 more condition	187	16.0	[13.3, 19.1]	97	9.8	[7.6, 12.6]	59	7.2	[5.1, 10.0]

Note: *N* = 3,025. PTSD = posttraumatic stress disorder.

The Pearson correlation coefficients (*p* < .001) for PTSD with depression, PTSD with anxiety, and depression with anxiety were slightly higher among the 1990s IDPs (.41, .39, .53, respectively) than the 2008 IDPs (.37, .30, .44, respectively) and returnees (.33, .35, .49, respectively). The characteristics associated with the three disorders and then their comorbidity are shown in Tables3 and 4, respectively. These highlight that being a returnee was associated with significantly lower rates above the cut score when compared with the reference population of 1990s IDPs, after adjusting for the influence of other factors, with the odds of being above from a one third lower probability for PTSD (odds ratio [*OR*] = 0.63), and around two thirds for depression (*OR* = 0.33), and around half for any one of the three (*OR* = 0.52). The 2008 IDPs were also associated with a significantly lower probability of depression (*OR* = 0.54) and having ≥1 condition (*OR* = 0.67) than 1990s IDPs. The same models were also run comparing returnees with a reference category of 2008 IDPs (i.e., excluding 1990s IDPs); these also showed a significantly lower probability among returnees compared to 2008 IDPs for PTSD (*OR* = 0.67), depression (*OR* = 0.61), anxiety (*OR* = 0.64), any condition (*OR* = 0.60), and ≥ one condition above the cut score (*OR* = 0.67).

**Table 3:** Regression Analyses of Characteristics Associated With Individual Presumed Mental Disorders

	PTSD				Depression				Anxiety			
Variable	*n*	%	*OR*	95% CI	*n*	%	*OR*	95% CI	*n*	%	*OR*	95% CI
Displacement status (all)^a^												
1990s	317	27.1	1.00		223	18.7	1.00		155	13.0	1.00	
2008	226	22.9	1.00	[0.80, 1.24]	99	9.9	0.54	[0.41, 0.71]**	92	9.2	0.81	[0.60, 1.09]
Returnees	140	17.0	0.63	[0.49, 0.81]**	60	7.2	0.33	[0.23, 0.45]**	55	6.6	0.51	[0.36, 0.73]**
Displacement status (2008 & returnees)^a^												
2008	226	22.9	1.00		99	9.9	1.00		92	9.9	1.00	
Returnees	140	17.0	0.67	[0.52, 0.87]*	60	7.2	0.61	[0.42, 0.88]*	55	7.2	0.64	[0.44, 0.93]*
Sex												
Men	188	18.7	1.00		116	11.4	1.00		77	7.6	1.00	
Women	507	25.6	1.67	[1.33, 2.09]**	308	15.4	1.50	[1.13, 1.98]**	240	12.0	1.79	[1.31, 2.46]**
Age (years)												
18–39	139	12.1	1.00		77	6.6	1.00		66	5.7	1.00	
40–59	246	24.1	1.80	[1.37, 2.37]**	153	14.7	2.00	[1.41, 2.85]**	113	10.9	1.56	[1.07, 2.29]*
60+	302	37.0	3.07	[2.33, 4.04]**	189	22.9	2.89	[2.02, 4.14]**	135	16.3	2.11	[1.43, 3.10]**
Education												
Higher education	106	16.9	1.00		63	9.9	1.00		46	7.2	1.00	
Secondary school	502	24.1	1.52	[1.14, 2.03]**	299	14.2	1.53	[1.07, 2.19]*	230	10.9	1.54	[1.04, 2.28]*
Primary/some secondary	90	32.8	2.02	[1.35, 3.03]**	65	23.4	2.68	[1.63,4.42]**	44	15.6	2.15	[1.24, 3.71]**
Trauma exposure^b^												
Lack of shelter	389	30.4	1.52	[1.23, 1.89]**	208	15.9			157	12.0		
Serious injury	174	37.0	1.66	[1.28, 2.16]**	112	23.4	1.57	[1.16, 2.13]**	88	18.3	1.71	[1.24, 2.37]**
Physical abuse	30	36.4			23	28.5	2.16	[1.10, 4.25]*	27	32.4	3.79	[2.07, 6.92]**
Witnessed murder, violence against stranger	88	46.9	2.12	[1.44, 3.11]**	54	28.7	1.58	[1.05, 2.40]*	40	21.4	1.62	[1.03, 2.53]*
Number of events^b^												
None	111	13.5	1.00		74	8.9	1.00		56	6.7	1.00	
1	164	20.3	1.57	[1.15, 2.14]**	96	11.8	1.26	[0.86, 1.85]	64	7.8	1.09	[0.71, 1.68]
2	153	23.9	1.80	[1.31, 2.48]**	90	13.9	1.35	[0.91, 2.00]	71	10.9	1.47	[0.96, 2.26]
3+	258	36.2	2.76	[2.02, 3.77]**	158	21.6	1.65	[1.14, 2.40]**	122	16.7	1.97	[1.30, 2.97]**
Household economic status												
Good/very good	208	14.4	1.00		102	7.0	1.00		78	5.3	1.00	
Bad/very bad	473	30.7	1.88	[1.50, 2.36]**	311	20.0	2.53	[1.89, 3.38]**	232	14.9	2.41	[1.74, 3.34]**
Community conditions												
Good/very good	231	19.6	1.00		125	10.5	1.00		100	8.4	1.00	
Average	210	20.7	1.13	[0.87,1.46]	127	12.4	1.35	[0.98, 1.87]	101	9.9	1.30	[0.91, 1.84]
Bad/very bad	244	30.8	1.71	[1.33, 2.20]**	163	20.2	1.89	[1.39, 2.57]**	112	13.9	1.52	[1.08, 2.14]*
	Pseudo *R*^2^ = .25 *p* < .001				Pseudo *R*^2^ = .25 *p* < .001				Pseudo *R*^2^ = .21 *p* < .001			

*NoteN* = 3,025. PTSD = posttraumatic stress disorder.

a Separate multivariate regression models run for the two displacement groupings. Regression results for other independent variables based on the first model (1990s internally displaced persons, 2008 internally displaced persons, and returnees).

b Separate multivariate regression models run for number of events and individual trauma events. Regression results for other independent variables based on number of events model. There were no significant differences (*p* < .05) for the results of the other independent variables between the two regression models. Referent category for trauma events was no exposure. Blank cells indicate where independent variables omitted after stepwise regression analysis.

* *p* < .05.

** *p* < .01.

**Table 4:** Regression Analyses of Characteristics Associated With Comorbidity

	Any condition				More than 1 condition			
Variable	*n*	%	*OR*	95% CI	*n*	%	*OR*	95% CI
Displacement (all groups)^a^								
1990s	415	34.8	1.00		187	16.0	1.00	
2008	282	28.3	0.90	[0.73, 1.10]	97	9.8	0.67	[0.50, 0.90]*
Returnees	173	20.7	0.52	[0.41, 0.66]**	59	7.2	0.40	[0.28, 0.57]**
Displacement (2008 & returnees)^1^								
2008 displaced	282	28.3	1.00		97	9.8	1.00	
Returnees	173	20.7	0.60	[0.47, 0.77]*	59	7.2	0.67	[0.46, 0.97]**
Sex								
Men	250	24.5	1.00		89	8.9	1.00	
Women	641	32.0	1.63	[1.33, 2.01]**	282	14.2	1.85	[1.36, 2.51]**
Age (years)								
18–39	202	17.4	1.00		52	4.6	1.00	
40–59	331	31.9	1.78	[1.39, 2.27]**	129	12.7	2.42	[1.62, 3.61]**
60+	349	42.2	2.44	[1.89, 3.16]**	184	22.6	4.23	[2.83, 6.31]**
Education status								
Higher education	139	21.9	1.00		51	8.2	1.00	
Secondary school	645	30.6	1.57	[1.21, 2.04]**	267	12.8	1.62	[1.11, 2.38]*
Primary/some secondary	111	39.8	2.22	[1.51, 3.27]**	55	20.0	2.34	[1.37, 3.99]**
Trauma exposure^b^								
Lack of shelter	466	35.7	1.25	[1.02, 1.52]*	190	14.8		
Serious injury	220	45.7	1.72	[1.34, 2.20]**	103	21.9	1.83	[1.33, 2.51]**
Physical abuse	45	54.4	2.48	[1.42, 4.31]**	23	27.8	2.71	[1.37, 5.35]**
Witnessed murder, violence against stranger	108	56.9	2.26	[1.55, 3.29]**	47	25.1		
Number of events^b^								
None	152	18.3	1.00		58	7.1	1.00	
1	204	31.3	1.41	[1.06, 1.86]*	87	10.8	1.49	[0.99, 2.24]
2	321	43.9	1.81	[1.35, 2.41]**	81	12.6	1.58	[1.03, 2.41]*
3+	277	18.9	2.55	[1.92, 3.37]**	140	19.6	1.97	[1.31, 2.97]**
Household economic status								
Good/very good	277	18.9	1.00		80	5.5	1.00	
Bad/very bad	598	38.4	2.05	[1.67, 2.52]**	281	18.3	2.68	[1.94, 3.68]**
Community conditions								
Good/very good	294	24.6	1.00		105	9.0	1.00	
Average	281	27.4	1.26	[0.99, 1.59]	116	11.4	1.46	[1.03, 2.06]*
Bad/very bad	304	37.8	1.70	[1.35, 2.16]**	142	17.9	2.00	[1.43, 2.78]**
	Pseudo *R*^2^ = .25 *p* < .001				Pseudo *R*^2^ = .29 *p* < .001			

*NoteN* = 3,025.

a Separate multivariate regression models run for the two displacement groupings. Regression results for other independent variables based on the first model (1990s internally displaced persons, 2008 internally displaced persons, and returnees)

b Separate multivariate regression models run for number of events and individual trauma exposure. Regression results for other independent variables based on number of events model. There were no significant differences (*p* < .05) for the results of the other independent variables between the two regression models. Referent category for trauma events was no exposure. Blank cells indicate where independent variables omitted after stepwise regression analysis.

* *p* < .05.

** *p* < .01.

There were a number of significant differences (*p* < .05) between the three population groups in exposure to traumatic events (Table1). These included a greater proportion of IDPs from both the 1990s and 2008 conflicts reporting having experienced a lack of shelter and being directly caught in a combat situation than the returnees. The 1990s IDPs reported significantly higher levels than 2008 IDPs and returnees of serious injury, witnessing the murder or violent acts against family/friends and strangers, and the death of family member/close friend during conflict/displacement.

Trauma-exposure events involving lack of shelter, serious injury, physical abuse, and witnessing a murder or violent acts against a stranger were commonly associated with the disorders and their comorbidity, as were cumulative trauma events. Other significant characteristics associated with being above the cut on one or more included sex (women), older age, and bad/very bad household economic situation and community conditions (Tables3 and 4). The mean functional disability score for 1990s IDPs (14.61) was significantly higher (i.e., worse disability) than the 2008 IDPs (8.99) and returnees (9.37). The other characteristics associated with worse disability are provided in Table5. The mental disorders all showed significant associations with worse disability, with more than one disorder having the strongest association. Sex, older age, and having an existing disability/long-term illness were also all significantly associated with higher disability.

**Table 5:** Regression Analyses of Characteristics Associated With Outcome of Functional Disability by Displacement Status

	Combined population		1990s Displaced		2008 Displaced		Returnees	
Variable	*B*	95% CI	*B*	95% CI	*B*	95% CI	*B*	95% CI
Mental disorders								
PTSD	6.38	[6.03, 6.74]**	7.57	[6.99, 8.15]**	5.21	[4.61, 5.81]**	6.19	[5.54, 6.84]**
Depression	9.67	[9.19, 10.15]**	9.32	[8.61, 10.03]**	11.07	[10.17, 11.97]**	8.08	[7.07, 9.08]**
Anxiety	6.25	[5.72, 6.77]**	6.68	[5.87, 7.49]**	5.84	[4.94, 6.74]**	4.36	[3.29, 5.43]**
Any condition	10.57	[10.25, 10.89]**	12.42	[11.89, 12.95]**	9.73	[9.18, 10.28]**	8.04	[7.46, 8.63]**
> 1 condition	15.91	[15.46, 16.36]**	17.12	[16.44, 17.79]**	14.62	[13.78, 15.47]**	13.76	[12.86, 14.66]**
Sex								
Women	2.29	[2.01, 2.56]**	3.43	[2.95, 3.90]**	.60	[0.11, 1.09]*	2.93	[2.46, 3.40]**
Age (years)								
40–59	3.08	[2.77, 3.40]**	2.76	[2.22, 3.31]**	2.07	[1.51, 2.63]**	3.97	[3.45, 4.49]**
60+	11.49	[11.14, 11.83]**	9.89	[9.28, 10.49]**	11.42	[10.81, 12.03]**	12.65	[12.08, 13.23]**
Disability/long-term illness								
Yes	8.56	[8.25, 8.88]**	8.40	[7.88, 8.91]**	10.85	[10.26, 11.45]**	6.45	[5.94, 6.96]**
Displacement status								
2008	−3.13	[−3.44, −2.82]**						
Returnees	−2.62	[−2.95, −2.29]**						
	Adj *R^2^* = .36 *p* < .001		Adj *R*^2^ = .36 *p* < .001		Adj *R*^2^ = .36 *p* < .001		Adj *R*^2^ = .29 *p* < .001	

*NoteN* = 3,025. PTSD = posttraumatic stress disorder. Referent categories are no PTSD, no depression, no conditions (for any condition), no condition (for > 1 condition), men, age 18–39 years, no disability, 1990s displaced. Separate multivariate regression models used: (a) PTSD, depression and anxiety plus sex, age, and disability; (b) any condition plus sex, age, and disability; and (c) > 1 condition plus sex, age, and disability. The results for sex, age and disability/long-term illness and Adj *R*^2^ results shown in table are from Model a. Same process applied for each population group.

* *p* < .05.

** *p* < .01.

## Discussion

This study provides the first representative data on mental health of adult IDP populations in Georgia. We recorded levels of presumed PTSD, depression, and anxiety of 23.3%, 14.0%, and 10.4%, respectively, for the combined study sample. These are lower than the rates of depression (70%) and anxiety (73%) recorded in a previous study of elderly IDPs in Georgia (Johns Hopkins Bloomberg School of Public Health/Institute for Policy Studies, 2012), but older age was associated with mental disorders in our study. Levels of mental disorders reported among IDPs and refugees globally vary considerably, but estimated overall averages for PTSD and depression among conflict-affected civilian populations globally are around 30%; with the variances in prevalence between studies reflecting differences such as levels of exposure to traumatic events and daily stressors, time periods, population types, study sampling, instrument selection, and cutoffs (Porter & Haslam, 2005; Steel et al., 2009). This study also highlights a number of factors associated with the mental disorders, including sex, age, education status, trauma exposure (particularly cumulative exposure), and daily stressors such as low household income and poor community conditions; our findings reflect those from other studies of conflict-affected civilian populations (Miller & Rasmussen, 2010; Porter & Haslam, 2005; Steel et al., 2009).

Our findings show the persistence of PTSD symptoms, particularly among 1990s IDPs. They also indicate significantly better mental health among returnees than the 1990s IDPs. The higher rates of mental disorders among the displaced are consistent with previous research examining the influence of forced displacement on mental health (Porter & Haslam, 2005; Steel et al., 2009). Our findings contribute to the limited evidence globally on returnees, particularly as the existing research has focused on returned refugees rather than returned IDPs (Roth, Ekblad, & Agren, 2006; Toscani et al, 2007; von Lersner, Elbert, & Neuner, 2008).

The data do not provide a clear explanation for the variance in levels of mental disorders between the three study groups. Potential explanations include that the 1990s conflicts were much longer than the 2008 conflict and characterized by greater brutality (as evidenced by higher exposure to traumatic events such as witnessing murder and violence and suffering physical abuse). Mental disorders may also become entrenched over a sustained period of time when also coupled with lack of access to adequate care and treatment as appears common in Georgia (Makhashvili & van Voren, 2013). Ongoing impoverishment and poor living conditions may also exacerbate existing disorders such as PTSD or contribute to causing mental disorders such as depression and anxiety (Makhashvili, Tsiskarishvili, & Drožđek, 2010; Miller & Rasmussen, 2010). Further research is required on the persistence of mental disorders in long-term displaced populations and returnees and the effectiveness of interventions to address them.

We found quite high levels of comorbidity, with over 40% of respondents with a presumed having more than one. PTSD has been found to be associated with high levels of comorbidity in other settings (Ayazi et al., 2012; O'Donnell et al., 2004), but we found comorbidity rates among those suffering with depression and anxiety (≈ 80%) to be significantly higher than for those with PTSD. These findings highlight the need for comprehensive evidence-based approaches that recognise and treat multiple disorders. This study also provides evidence on how these mental disorders influence functional disability and this reinforces how improvements in mental health could substantially strengthen broader individual well-being, as well as social and economic well-being.

The persistence of mental disorders and their comorbidity suggests that the treatment gap for mental disorders among conflict-affected populations in Georgia may be large and leading to chronic disability. Our findings support the need for a scaled-up, comprehensive, and trauma-informed response to support the mental health of conflict-affected populations in Georgia. Given the protracted nature of the displacement in Georgia and its impact on mental disorders and functioning, the government of Georgia should seek to provide more durable long-term solutions, including strengthening socioeconomic conditions.

This study has a number of limitations. The cross-sectional design means that causation cannot be attributed, and the temporal relationship between risk factors and outcomes cannot be determined. As a result, reverse causality cannot be excluded for the more subjective risk factors (e.g., community conditions and household economic status). The lack of available data on the prevalence of mental disorders among the general population of Georgia also prevents comparisons with them. The study did not include IDPs hosted by relatives or friends or living independently away from the formal and informal settlements. The long recall period could increase potential recall bias for exposure to violent and traumatic events, particularly for the 1990s IDPs. The study did not assess respondents’ psychiatric history and functioning levels prior to their exposure to the conflicts and forced displacement, largely due to concerns over recall bias (Simon & VonKorff, 1995), but it is recommended that future studies should seek to assess these where possible. Lastly, although we provide data on the validity and reliability of the study instruments with the study population, these instruments and their cutoffs were not comprehensively normed and so our data cannot strictly be said to measure rates of disorder rather than a certain level of symptoms.

In conclusion, this study highlights the persistence of PTSD among conflict-affected persons in Georgia and the need for a comprehensive approach to tackling trauma-related and common mental disorders and functioning.
